# Field efficacy of a biopesticide and a predatory mite for suppression of *Scirtothrips dorsalis* (Thysanoptera: Thripidae) in strawberry

**DOI:** 10.1093/jee/toae144

**Published:** 2024-06-28

**Authors:** Sriyanka Lahiri, Gagandeep Kaur, Allan Busuulwa

**Affiliations:** Gulf Coast Research and Education Center, Department of Entomology and Nematology, University of Florida, Wimauma, FL 33598USA; Gulf Coast Research and Education Center, Department of Entomology and Nematology, University of Florida, Wimauma, FL 33598USA; Crop Protection Research and Development, Corteva Agriscience, 9330 Zionsville Rd, Indianapolis, IN, 46268USA; Gulf Coast Research and Education Center, Department of Entomology and Nematology, University of Florida, Wimauma, FL 33598USA

**Keywords:** Captiva Prime, radiant, biocontrol, botanicals, chilli thrips

## Abstract

Chilli thrips, *Scirtothrips dorsalis* Hood (Thysanoptera: Thripidae) has emerged as a severe invasive pest of strawberry *Fragaria × ananassa* Duchesne in the United States. The objective of this study was to assess the field efficacy of a biopesticide and thrips predator, *Amblyseius swirskii* Athias-Henriot for *S. dorsalis* management in field grown strawberry compared to synthetic insecticide applications that are current industry standard (spinetoram) conducted at UF/IFAS GCREC, FL during 2021–2022 and 2022–2023 in a 2-year field study. The following treatments were applied in the field: (1) biopesticide, capsicum oleoresin extract + garlic oil + canola oil application at maximum label rate; (2) predatory mite of thrips, *A. swirskii* released at 30 predators per plot; (3) spinetoram applied at maximum label rate; and (4) biopesticide applied 24 h before release of *A. swirskii*. A control plot with no insecticide or predatory mite releases was maintained. Results show that the capsicum extract can be used for management of *S. dorsalis*, especially during the latter stages of strawberry field season when resistance to spinetoram is high. The field performance of *A. swirskii* was variable and extensive research is needed to highlight factors affecting field performance of predatory mites for thrips management.

## Introduction

Since 2015, chilli thrips, *Scirtothrips dorsalis* Hood (Thysanoptera: Thripidae) has emerged as a severe invasive pest of strawberry *Fragaria × ananassa* Duchesne (Rosaceae) in Florida, USA, and has spread rapidly across other southeastern and northeastern United States (Kaur and Lahiri 2022, Kumar et al. 2023). Originally from Southeast Asia, the worldwide economic impact of *S. dorsalis* has been recorded from agricultural crops (Tatara 1994, Ebratt et al. 2018). It is a polyphagous pest and has developed resistance to several insecticides (Muraoka 1988, Kaur et al. 2023). The rapid development of *S. dorsalis* during a typical strawberry season can be attributed to their capacity to produce an estimated 50 eggs per female, and minimum and maximum temperature for development of 9.7 °C and 32 °C, respectively (Tatara 1994).

Commercially available biopesticide formulation of capsicum oleoresin extract + garlic oil + canola oil and predatory mite *Amblyseius swirskii* Athias-Henriot (Arachnida: Phytoseiidae) are effective management tools for *S. dorsalis* in greenhouse strawberry production (Lahiri and Yambisa 2021). Although biopesticides such as those derived from plants (botanicals) or biocontrol agents such as predators of thrips can provide much needed integrated pest management, their field efficacy is not well understood for open-field *S. dorsalis* management in strawberry crop. Therefore, the study was to assess the field efficacy of a biopesticide and *A. swirskii* for *S. dorsalis* management in field grown strawberry. The hypothesis was that both the biopesticide and biocontrol agent will show equal field efficacy compared to conventional insecticide treated strawberry crop.

## Materials and Methods

During both years of field study (2021–2022 and 2022–2023), field experimental plots were established and maintained at University of Florida, Gulf Coast Research and Education Center, Wimauma, Florida, USA (27.76696, −82.22701), as described in Montemayor et al. (2022). The beds were prepared following local commercial strawberry production standards. Raised beds (20-cm high) covered with black virtually impermeable plastic film (Total Blockade TIF Mulch, Berry, Morgantown, PA, USA), were prepared in August every year and fumigated with a soil fungicide/nematicide (Telone C-35, 280 L/ha, Dow AgroSciences). A standard nitrogen fertilizer was also incorporated into the soil during bed preparation at 2.2 kg/ha rate. Each bed row had a single drip tape running through the center and was hand-punched uniformly so that 2 rows of strawberry plants could be planted at a depth of 10-cm (4-inch) with between-row plant and within-row plant distance at 30-cm (1 ft). Adjacent strawberry raised beds were 121.92-cm (4 ft) apart.

Bare-root strawberry transplants of the cultivar ‘Florida Brilliance’ (US Patent PP30,564) were obtained from a nursery (G.W. Allen Nursery Ltd., Nova Scotia, Canada) and transplanted by hand in mid-October, during both years. Strawberry plots (4.57-m long and 3.04 m buffer) with 20 plants per plot were established in this way and received overhead irrigation for 10 days post-transplanting. An early season herbicide treatment with flumioxazin (Chateau® Herbicide SW, 210.16 g/ha, Valent USA, LLC Agricultural Products) around raised bed borders as well as a preventative treatment with *Bacillus thuringiensis* subsp. *kurstaki*, strain ABTS-351 (DiPel® DF, 2.24 kg/ha, Valent USA, LLC) for armyworms *Spodoptera* spp. (Lepidoptera: Noctuidae) on the vegetative growth stage of strawberry was done. The following treatments were applied in the field: (1) commercial biopesticide, capsicum oleoresin extract (7.60%) + garlic oil (23.40%) + canola oil (55%) + other unknown ingredients (14%) (Captiva Prime®, Gowan Company) application at maximum label rate of 2.34 L/ha (2 pints/a); (2) predatory mite, *A. swirskii* (BioBee USA, Salisbury, MD) released at 30 predators per plot; (3) conventional insecticide, spinetoram (Radiant®SC) applied at maximum label rate of 0.73 L/ha (10 fl oz./A); and (4) biopesticide (Captiva Prime®) applied 24 h before release of *A. swirskii*. A control plot with no insecticide or predatory mite releases was maintained. Predators were released by hand, wherein, 30 individuals were carefully collected in 5-ml micro-centrifuge tubes, which were attached to a strawberry plant in the center of the plot, with twist ties and lid was opened. According to Schoeller et al. (2020), *A. swirskii* prefers larval *S. dorsalis* compared to adults and can consume 4.6–6.3 larvae/day compared to 1.6–1.7 adults/day. The experiment was arranged in a randomized complete block design with 5 replications and the plots were arranged in a checker-board pattern to avoid treatment effects in adjacent beds of strawberry plots. Weekly sampling of 6–8, random, young, and fully expanded strawberry trifoliates and plant damage rating specific to *S. dorsalis* feeding was conducted during the duration of the study in both years between Dec and Feb following protocol described in Lahiri and Yambisa (2021). Briefly, the following leaf damage parameters were used: 0 = no feeding damage; 1 = base of trifoliate mid-rib vein shows dark necrotic tissue; 2 = dark necrotic tissue appears spreading from entire leaf mid-rib to all lateral veins; 3 = in addition to leaf veins turning dark in color, leaf edges start curling upwards; 4 = 80% of trifoliate loosing green color and turning dry and crinkled; and 5 = dead plant due to no recovery from leaf tissue damage. Treatments were initiated as soon as average damage rating per plot reached 1. Sampled trifoliates per plot were pooled into 1 plastic resealable bag and transported to the laboratory. Ethanol (70%) was poured into these bags to completely submerge the trifoliate and shaken vigorously for ~5 s. This ethanol wash was then collected in a gridded, square, polystyrene petri plate (Fisher Scientific, Hampton, NH, USA) to count *S. dorsalis* life stages. Marketable fruit yield data was collected by conducting hand-harvesting once a week from all 20 plants per plot, followed by fruit weighing (g). Fruits damaged by *S. dorsalis* feeding turned bronzed and cracked and were discarded during the plot clean-up process and only undamaged fruits were weighed to assess marketable fruit yield.

## Data Analysis

The effect of the various treatments in plots on *S. dorsalis* suppression was assessed using a mixed model’s analysis of variance (ANOVA, PROC MIXED, SAS Enterprise Guide v. 6.1, SAS Institute Inc. 2015. SAS/STAT® 14.1 User’s Guide. Cary, NC) approach. The significant differences were separated using the methods of Tukey (Tukey 1953) (α ≤ 0.05).

## Results

### Year 1

#### Chilli thrips count

No impact of spinetoram, capsicum extract, and *A. swirskii* treatments was evident on the *S. dorsalis* adult and larval density in the strawberry field in 2021–22 (*F* = 2.07; *df* = 4, 91; *P* = 0.0918; *F* = 2.51; *df* = 4, 91; *P* = 0.0471, respectively). The season-long mean (±*SE*) adult and larval *S. dorsalis* count across all treatments and control strawberry plots was 4.11 (±0.49) and 8.85 (±0.88), respectively.

#### Yield

**Fig. 1.**
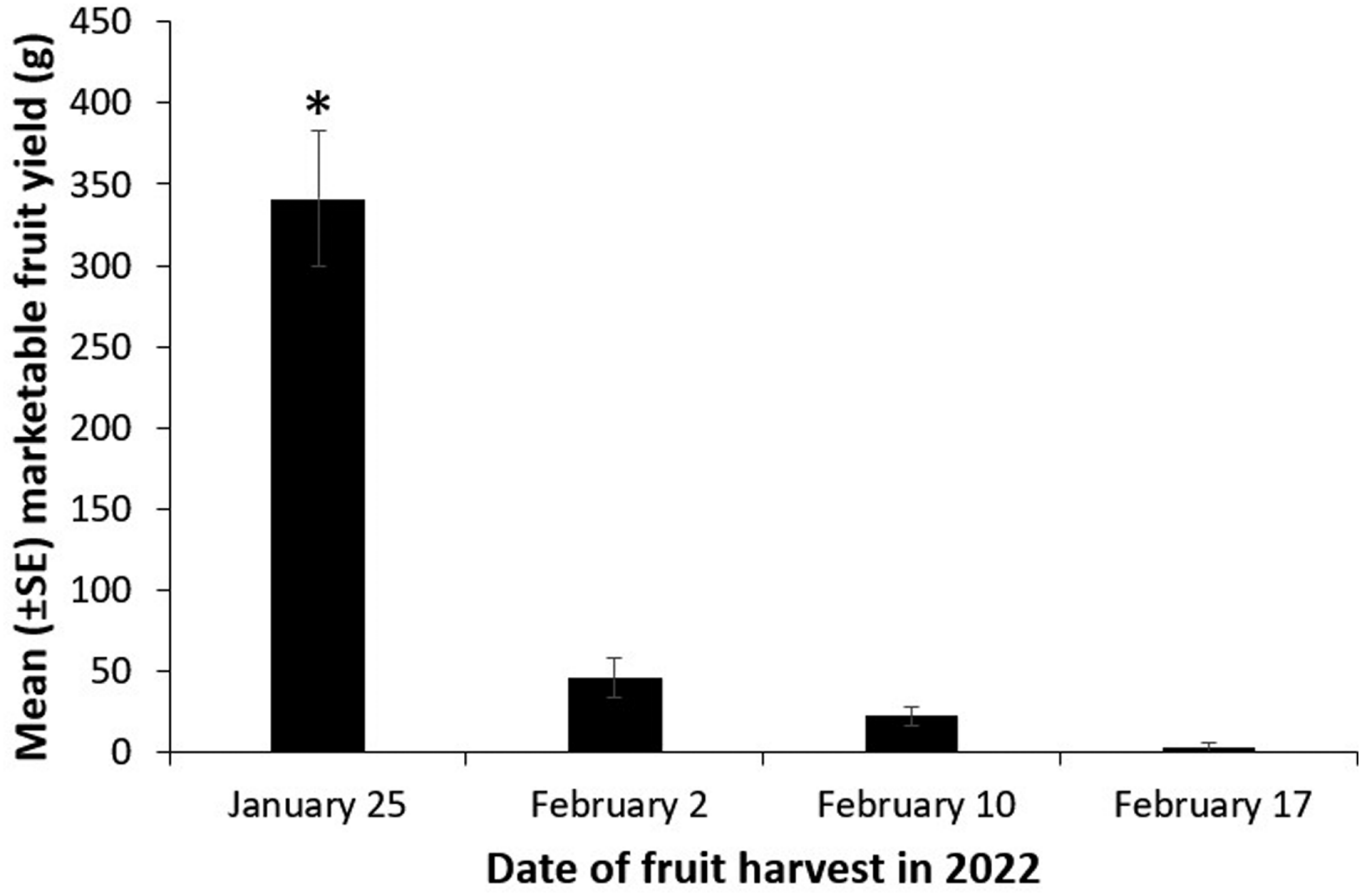
Mean (±*SE*) marketable fruit yield (g) of strawberry on 4 harvest dates in Wimauma, FL in 2022 with 20 ‘Florida Brilliance’ plants per plot. Asterisk represents significantly high yield. (ANOVA, Proc Mixed followed by Tukey’s HSD, α = 0.05, SAS Institute Inc., Cary, NC).

There was no improvement in the marketable fruit yield of strawberry after application of any of the treatments compared to the nontreated plants (*F* = 0.53; *df* = 4, 76; *P* = 0.7117). Although there was no interaction effect of date of harvest and treatment, (*F* = 0.32; *df* = 12, 76; *P* = 0.9833), there was a significant main effect of date of harvest on fruit yield (*F* = 50.75; *df* = 3, 76; *P* < 0.001; Fig. 1).

#### Damage rating

Strawberry plants that were treated with spinetoram [1.76 (±0.11)], capsicum extract [1.75 (±0.12)], and *A. swirskii* [1.44 (±0.08)], sustained much lower feeding damage from *S. dorsalis* when compared to those treated with the combination of capsicum extract + *A. swirskii* [1.88 (±0.12)], and control (*F* = 6.97; *df* = 4, 476; *P* < 0.0001). The damage sustained by all the plants was rated between 1 and 2 this season with control plants showing a rating of 2.26 (±0.15). The damage rating varied significantly with date of sampling during the month of Feb (*F* = 3.46; *df* = 3, 476; *P* = 0.0164). However, there were no interaction effects between the treatments and the date of sampling (*F* = 1.11; *df* = 12, 476; *P* = 0.3501). A higher plant damage rating of 2.04 (±0.11) was visible on 17th Feb 2022 (4th week after treatment) when compared to the damage rating of 1.6 (±0.10) on 10th Feb 2022 (3rd week after treatment). This was the maximum visible damage on strawberry plants recorded this year.

### Year 2

#### Chilli thrips count

**Fig. 2.**
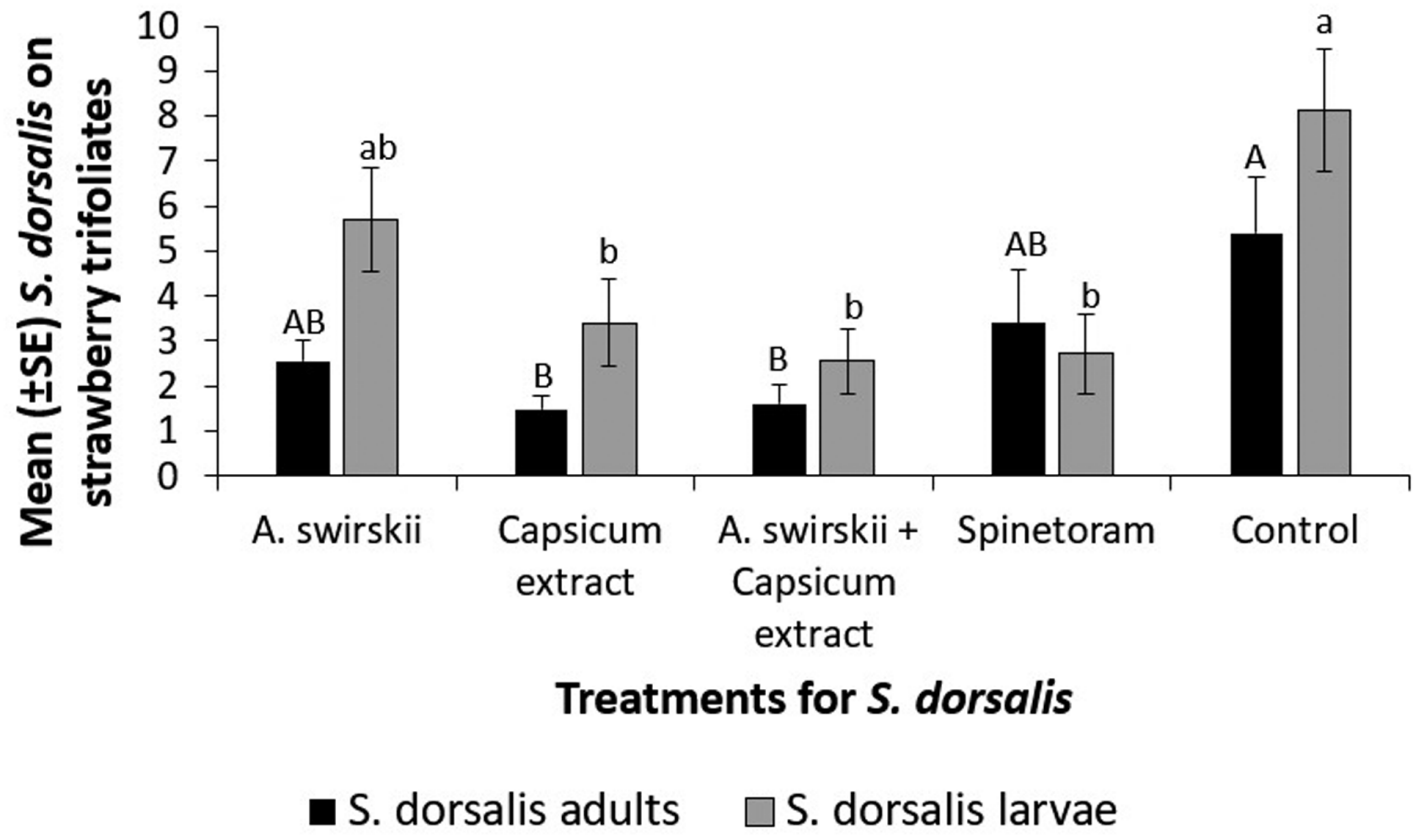
Mean (±*SE*) *S. dorsalis* adult and larval count on strawberry trifoliates from plants treated with one of the following: predatory mite *A. swirskii*, botanical biopesticide capsicum extract, a combination of capsicum extract and *A. swirskii*, and synthetic insecticide spinetoram. These treatments were compared to a non-treated control plot (ANOVA, Proc Mixed followed by Tukey’s HSD, α = 0.05, SAS Institute Inc., Cary, NC). Each treatment and control had twenty ‘Florida Brilliance’ plants per plot, arranged in a randomized complete block design and replicated 5 times.

A significant impact of the various insecticidal treatments was evident on both *S. dorsalis* adult and larvae in the strawberry field in 2022–23 (*F* = 4.71; *df* = 4, 116; *P* = 0.0015; *F* = 6.65; *df* = 4, 116; *P* < 0.0001, respectively; Fig. 2). In case of *S. dorsalis* adults, capsicum extract, and the combination of capsicum extract + *A. swirskii* contributed to significant pest suppression when compared with the control plants. However, there was no difference in adult found in plants treated with spinetoram and only *A. swirskii* when compared with non-treated control plants.

In the case of *S. dorsalis* larvae, all 3 treatments, that is capsicum extract, the combination of capsicum extract + *A. swirskii*, and spinetoram, significantly suppressed pest density compared to the control plants. Plants treated with only *A. swirskii* and the control plants had the same number of *S. dorsalis* larvae.

### Yield

**Fig. 3.**
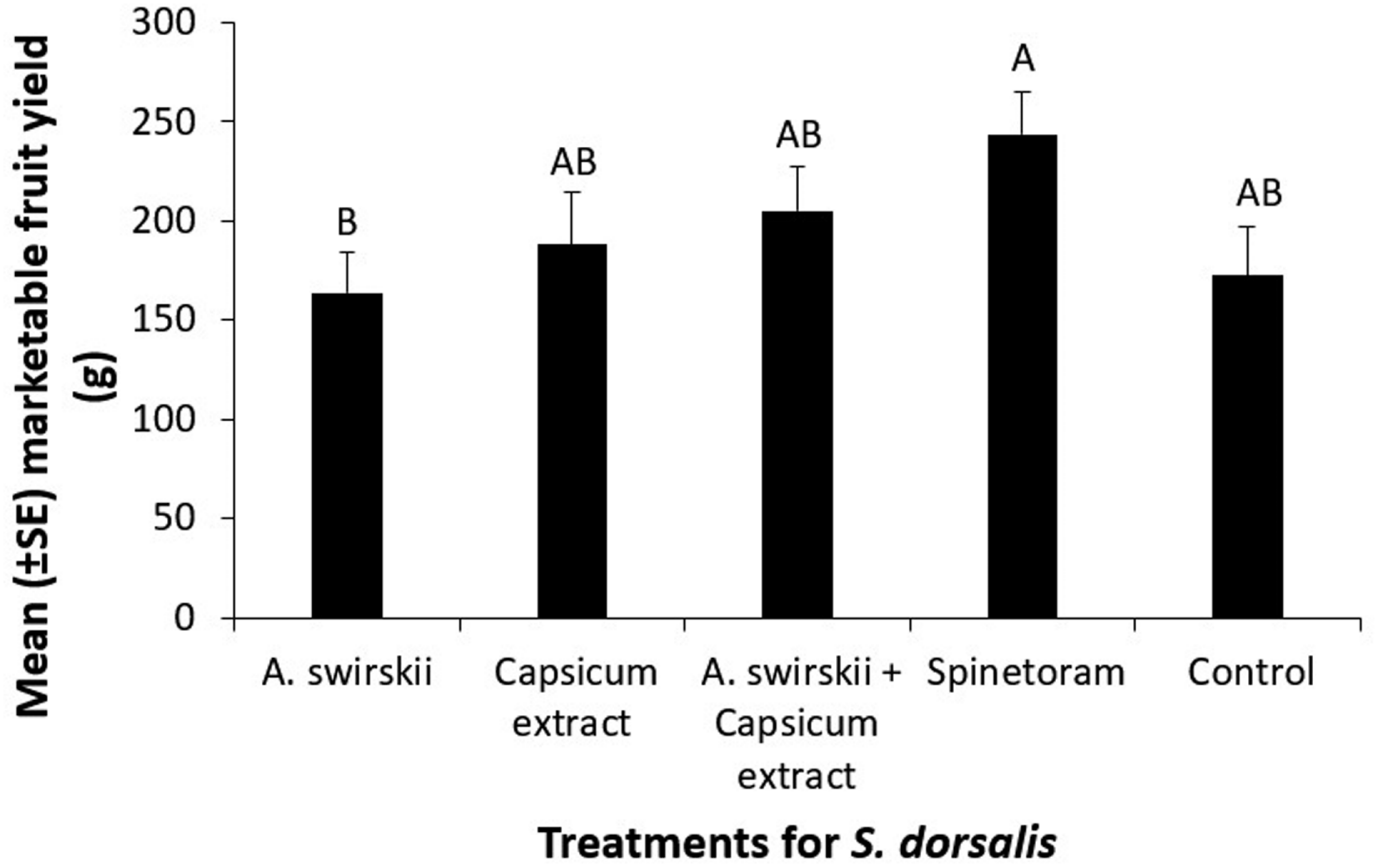
Mean (±*SE*) marketable fruit yield (g) of strawberry from plants treated with one of the following: predatory mite *A. swirskii*, botanical biopesticide capsicum extract, a combination of capsicum extract and *A. swirskii*, and synthetic insecticide spinetoram. These treatments were compared to a non-treated control plot (ANOVA, Proc Mixed followed by Tukey’s HSD, α = 0.05, SAS Institute Inc., Cary, NC). Each treatment and control had twenty ‘Florida Brilliance’ plants per plot, arranged in a randomized complete block design and replicated 5 times.

The marketable strawberry fruit yield in plants treated with spinetoram was significantly higher compared to plants treated with *A. swirskii* (*F* = 2.58; *df* = 4, 96; *P* = 0.0421; Fig. 3). Additionally, plants treated with the capsicum extract or the combination of capsicum extract + *A. swirskii* achieved similar fruit yield as the spinetoram treated plants.

**Fig. 4.**
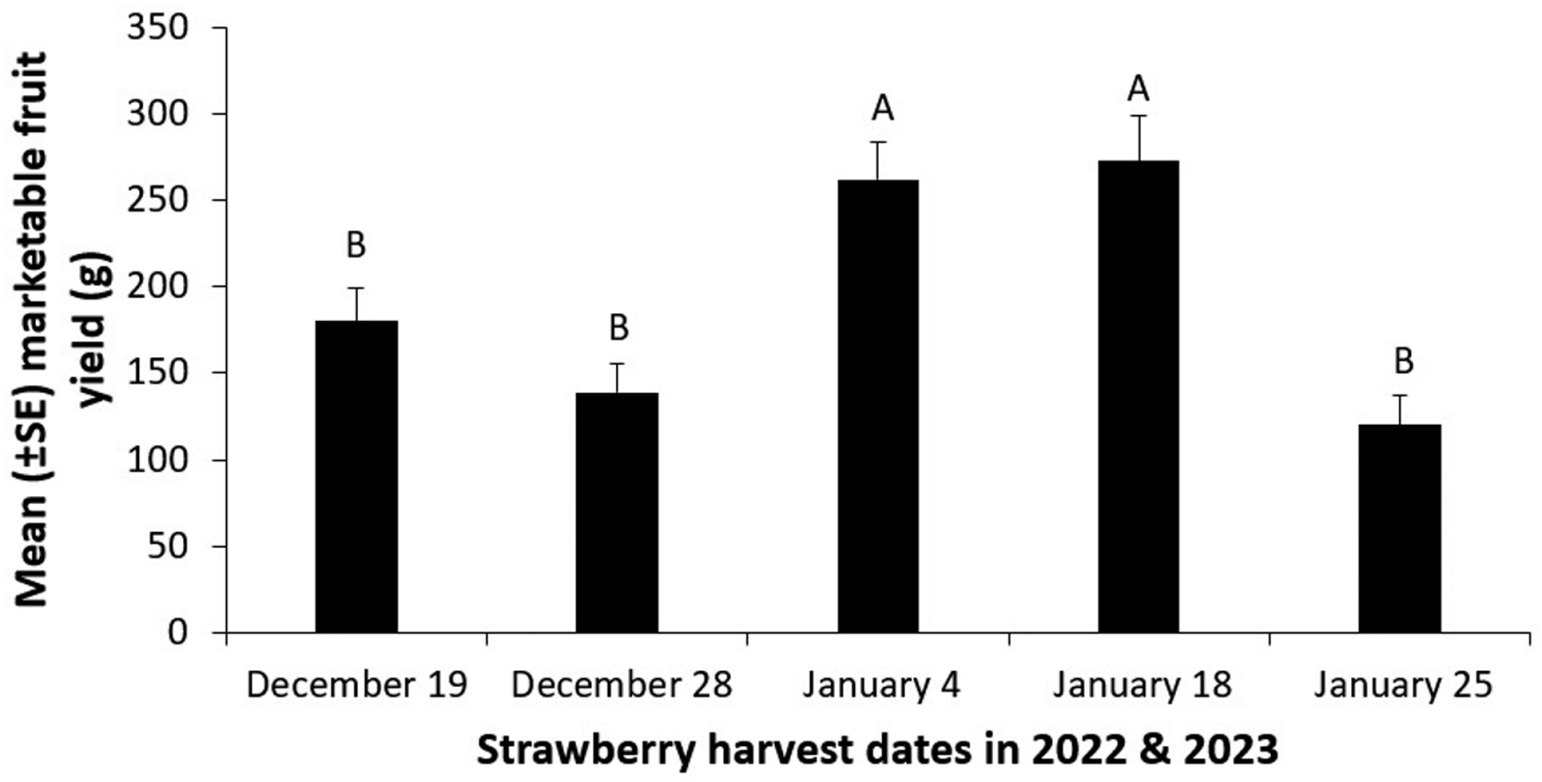
Mean (±*SE*) marketable fruit yield (g) of strawberry on 5 harvest dates in Wimauma, FL in 2023 (ANOVA, Proc Mixed followed by Tukey’s HSD, α = 0.05, SAS Institute Inc., Cary, NC).

The date of fruit harvest also had a significant effect on fruit yield (*F* = 12.58; *df* = 4, 96; *P* < 0.0001). The harvest during the 1st and 2nd week of January 2023 from the 20 plant plots was significantly higher compared to that in the 3rd and 4th weeks of December 2022 and 4th week of January 2023 (Fig. 4). However, there were no interaction effects between date of fruit harvest and treatments (*F* = 0.78; *df* = 16, 96, *P* = 0.7086).

### Damage Rating

The plants treated with spinetoram displayed significantly high damage rating [2.34 (±0.14)] compared to the control plants [2.02 (±0.09)], whereas no such distinction could be elucidated among the other treatments (*F* = 2.53; *df* = 4, 971; *P* = 0.039). The season-long average damage rating in strawberry plots treated with *A. swirskii*, capsicum extract, and the combination of these 2 was 2.2 (± .11), 2.2 (±0.12), and 2.2 (±0.13), respectively. There was a significant main effect of date of sampling (*F* = 426.49; *df* = 4, 971; *P* < 0.0001), and an interaction effect of date of sampling and treatments (*F* = 12.77; *df* = 16, 971; *P* < 0.0001). The damage rating sustained by the infested plants was much higher during the last 2 sampling dates during 18th and 25th Jan 2023 [3.76 (±0.08)], compared to the earlier 2 sampling dates on 19th and 28th Dec 2022 [1.02 (±0.07); 1.43 (±0.08), respectively], and January 4th 2023 [1.01 (±0.07)]. The damage ratings on December 19th and January 4th were not statistically different.

## Discussion

The results of 2-years of field study show the importance of increasing reliance on biopesticides for the management of thrips. It was specifically highlighted that the botanical based biopesticides can function as valuable rotation products with conventional insecticides for invasive thrips pest management in strawberry. The limitations of using biocontrol agents, such as *A. swirskii*, was also highlighted in the field study.

The average plant damage caused by *S. dorsalis* feeding was low, therefore, the impact of the 3 rescue treatments (biopesticide, predatory mite, spinetoram) was unclear during 2021–22. This year, the date of harvest was the most important factor in affecting marketable fruit yield and *S. dorsalis* infestation was not a key factor in influencing crop yield. Rather, the overarching factor was the agronomic trait of the strawberry cultivar grown for the experiment. In this case, it was Florida Brilliance, which is a short-day strawberry cultivar bred for producing the bulk of fruits earlier during the season to capitalize on the demand for fresh strawberry in the markets (Whitaker et al. 2019).

Even with higher visible thrips damage on the plant, the fact that Florida Brilliance experienced similar fruit yield and pest numbers across all treatments, points to the capability of host plant tolerance/resistance in this cultivar. Examples of host plant resistance in certain strawberry cultivars to other arthropod pests include western flower thrips, *Frankliniella occidentalis* (Pergande) (Thysanoptera: Thripidae), two spotted spider mite, *Tetranychus urticae* Koch (Arachnida: Tetranychidae), strawberry mite *Phytonemus pallidus* spp. *Fragariae* (Banks) (Acari: Tarsonemidae), strawberry blossom weevil, *Anthonomus rubi* (Herbst) (Coleoptera: Curculionidae), and European tarnished plant bug, *Lygus rugulipennis* Poppius (Hemiptera: Miridae) (Schuster et al. 1980, Labanowska et al. 2004, Rahman et al. 2010). The presence of strawberry host plant resistance and/or tolerance to *S. dorsalis* needs to be further investigated. The capsicum extract and *A. swirskii* reduced feeding damage on strawberry plants, when used alone. No synergistic effect of combining capsicum extract + *A. swirskii* was evident in this field study.

Field observations during year 2 of this study were starkly different, perhaps owing to higher overall *S. dorsalis* feeding damage. Since *S. dorsalis* numbers were assessed during January–February during both years of the field study, the insecticide resistance status of *S. dorsalis* during that time of the strawberry season could have played a role. In fact, Kaur et al. (2023) have shown that *S. dorsalis* become highly resistant to several insecticide active ingredients such as bifenthrin, spinetoram, cyantraniliprole, and acetamiprid during January–February after receiving several insecticidal treatments since the beginning of the field season in October. This may be the leading cause of why spinetoram treated plots had the same *S. dorsalis* pest pressure as control plots, in this study.

The predatory mite, *A. swirskii*, was ineffective in suppressing *S. dorsalis* adults also. This is not surprising as *A. swirskii* has been shown to be an effective *S. dorsalis* larval predator rather than feeding on adults. In a greenhouse potted strawberry plant study, *A. swirskii* effectively suppressed *S. dorsalis* larval density for 21 days of experimental observation period, whereas the capsicum extract significantly suppressed the adult *S. dorsalis* only for 7 days (Lahiri and Yambisa 2021). In this greenhouse study, the efficacy of *A. swirskii* to suppress *S. dorsalis* larvae rivaled that of spinetoram treated strawberry plants. However, in the current field study, capsicum extract was effective in causing sustained *S. dorsalis* suppression, and a synergistic effect on *A. swirskii* was also evident. The larval *S. dorsalis* population was far more susceptible to the capsicum extract and combination of capsicum extract + *A. swirskii* mites, as well as spinetoram, in this field study. Unfortunately, the biocontrol agent, *A. swirskii*, was ineffective in field suppression of *S. dorsalis* larval populations. Based on this finding, it can be concluded that the capsicum extract based biopesticide can be used for management of *S. dorsalis* populations, especially during the latter stages of strawberry field season when resistance to synthetic insecticides such as spinetoram is high and impact of chemical control is low.

The field performance of *A. swirskii* may be variable. Extensive research is needed to highlight factors affecting field performance of predatory mites for thrips management in open-fields. Possible factors leading to failure of *A. swirskii* to suppress field populations of larval *S. dorsalis* include a lack of establishment due to poor health of lab reared individuals, lack of familiarity among the lab reared predators with the prey *S. dorsalis*, environmental conditions, and exposure to commonly used fungicides. Kütük et al. (2016) reported a failure of *A. swirskii* to suppress greenhouse populations of *F. occidentalis* on flowering eggplant *Solanum melongena* L. (Solanaceae) in Turkey due to various factors such as leaf trichome density and exudates, and pollen amount and fertility of eggplant flowers, even though mean winter temperature within the plastic greenhouse always remained above 11.3 °C; developmental threshold of *A. swirskii* (Lee and Gillespie 2011). Ersin et al. (2020) reported that *A. swirskii* larvae were most sensitive to insecticide and fungicide applications, where along with insecticide sulfoxaflor, fungicides including isopyrazam, propamocarb + fluopicolide, ametoctradin + dimethomorph, and mandipropamid caused significant mortality in *A. swirskii* eggs, adult female, and larvae. In fact, our research plots received weekly applications of various fungicides including N-trichloromethylthio-4-cyclohexane-1,2-dicarboximide, mefenoxam, tetramethylthiuram, and cyprodinil + fludioxonil, which may have contributed to decline in the density of *A. swirskii*. Since *A. swirskii* population assessment was not done during this field study, a clear justification regarding a lack of population establishment of biocontrol agents cannot be made. Lately, genetic improvement of biological control agents to overcome limitations such as pesticide compatibility, prey-density dependence, and extreme environmental conditions is being viewed as the next step for widespread application of biocontrol agents (Bielza et al. 2020).

Since fruit yield in strawberry plots receiving either spinetoram or capsicum extract rescue treatments improved significantly, capsicum extract is a reliable biopesticide that can be used for *S. dorsalis* pest management in strawberry, even in situations where spinetoram resistance in *S. dorsalis* is high. The fact that marketable strawberry fruit yield was high in spinetoram treated plots despite having higher *S. dorsalis* adult populations when compared to capsicum extract treated plants, points to tolerance in this strawberry cultivar during this year also.

The overall conclusion from this field study is that the capsicum extract based biopesticide can provide reliable *S. dorsalis* suppression in strawberry crops, which is highly relevant for organic and conventional strawberry growers having sustained difficulty with resistant *S. dorsalis* field populations. Although there is variability in performance of biocontrol agent *A. swirskii* under open-field conditions, corrective measures may be taken to improve resilience of *A. swirskii* for *S. dorsalis* management in strawberry. From a practical perspective, release of *A. swirskii* after a few days of fungicide or insecticide treatment may be most beneficial, keeping residual activity periods of pesticides in mind. Additionally, if fungicides/insecticides/miticides had to be applied to the crop while predatory mites were present, a second augmentative release of these biocontrol agents will be warranted. Also, host plant resistance has tremendous potential in playing an important role in the integrated pest management of *S. dorsalis*.
